# Oral health related quality of life of oral cancer patients treated with radiotherapy alone or with chemotherapy in a tertiary referral centre in Sri Lanka

**DOI:** 10.1186/s12903-023-02854-x

**Published:** 2023-03-19

**Authors:** Shamini Kosgallana, Prasanna Jayasekara, Prasad Abeysinghe, Ratilal Lalloo

**Affiliations:** 1 Ministry of Health, Colombo, Sri Lanka; 2 School of Dentistry, University of Queensland, Queensland, Australia; 3 Research & Surveillance Unit, Institute of Oral Health, Maharagama, Sri Lanka; 4 Apeksha Hospital, Maharagama, Sri Lanka

**Keywords:** Oral cancer, Oral health related quality of life, Radiotherapy, Chemotherapy, Modified EORTC QLQ-OH15

## Abstract

**Background:**

Oral cancer is the number one cancer among males in Sri Lanka. Radiotherapy is a common treatment modality for oral cancer, but this can affect oral health related quality of life (OHRQOL). This study assessed the OHRQOL and its changes from baseline to the last week of radiotherapy and three months post radiotherapy among oral cancer patients who received this treatment alone or with chemotherapy.

**Methods:**

A prospective longitudinal study was conducted among 90 oral cancer patients awaiting for radiotherapy alone or with chemotherapy. The modified Sinhala version of the European Organization for Research and Treatment of Cancer Quality of Life Questionnaire Oral Health Module (EORTC QLQ-OH15) was used to gather data related to OHRQOL before radiotherapy. Socio-demographic and clinical data were also recorded. The same cohort of patients were followed up and assessed their OHRQOL during the last week of radiotherapy and three months post radiotherapy. The Modified EORTC QLQ-OH15 assesses the OHRQOL under three domains namely ‘*Eating problem’*, ‘*Gum and speech problem’* and *‘Soreness’*, and one item named as *‘Teeth’.*

**Results:**

The majority of the sample (88%) was males. The anterior two-thirds of the tongue (40%) and buccal mucosa (22%) were the most common sites. The median scores of ‘*Eating problem’* domain at baseline, last week of radiotherapy and three months post radiotherapy were 20 (IQR = 6.7–33.3), 100 (IQR = 86.9–100.0) and 66.7 (IQR = 46.7–93.3) respectively. ‘*Gum and speech problem’* was higher during last week of radiotherapy (median, 50.0, IQR, 25.0-58.3) than three months post radiotherapy (median, 8.3, IQR, 0.0-33.3). The changes of OHRQOL between the time frames were statistically significant (p < 0.05). Baseline OHRQOL in relation to ‘*Gum and speech problem’* domain and *‘Teeth’* item was identified as an influential factor for OHRQOL during last week of radiotherapy.

**Conclusion:**

The OHRQOL of oral cancer patients who received radiotherapy alone or with chemotherapy had deteriorated from the baseline level to the last week of radiotherapy but then improved at three months post radiotherapy. The OHRQOL however did not return to the baseline level three months post radiotherapy. OHRQOL during the last week of radiotherapy was influenced by the OHRQOL at baseline, civil status and sites of metastasis.

## Introduction

Worldwide, 354,864 new oral cancer cases and 177,384 deaths due to oral cancer were identified in 2018. In the countries with low and medium on the Human Development Index, oral cancer incidence is 3rd common among males and the 7th most common cause of cancer deaths. More than 40% of head and neck cancer cases globally occur in the oral cavity [1]. South Asia was found to have the highest age standardize incident (9.65/100,000, 95%CI = 8.17–11.15/100,000) in 2019 and it was gradually increasing from 1990 in the region of Asia [2]. In 2019, oral cancer was the most common cancer among Sri Lankan men with an age standardized incidence rate of 19.1 per 100,000 male population. Further, it accounted for 15% of all cancers [3].

There are many different modalities available for treating cancers of the oral cavity. Surgery, radiotherapy (RT) and chemotherapy, alone or in combination are the most common treatments provided [4, 5]. Stage I and stage II oral cancers are highly curable by surgery or by RT giving equally good long term results, and function is better after RT than after surgery [6]. In addition, RT has shown 65 − 90% local control rate in moderately advanced oral cancers [7]. Although RT with or without chemotherapy alone is not practiced commonly to cure oral cancer, in situations where organ preservation is of concern, the patient’s failure to withstand the surgery, necessity to avoid cosmetic imperfections and maintain the functions, it is used with or without chemotherapy [8–10]. While these treatments are effective, they also have significant side effects. Side effects of RT specifically include mucositis (stomatitis), xerostomia (dry mouth), bacterial, fungal, or viral infections, dental caries, loss of taste, osteoradionecrosis, nutritional compromise, anorexia and malaise [11–13]. These complications affect the patients’ health related quality of life as well as oral health related quality of life (OHRQOL). The United States Surgeon General’s report on oral health defines OHRQOL as “a multidimensional construct that reflects (among other things) people’s comfort when eating, sleeping, and engaging in social interaction; their self-esteem; and their satisfaction with respect to their oral health” [14]. Patients with stage III and IV cancers have shown the poorest OHRQOL for swallowing, speech, social eating, reduced mouth opening, and dry mouth and it was same when treated with conventional RT compared to Intensity Modulated RT [15, 16]. In Sri Lanka more than 68% of oral cancers were first detected at stage III and IV [17]. Most of the patients were treated with the conventional cobalt RT as only limited number of linear accelerators with Intensity Modulated RT are available Sri Lanka [18].

Assessing OHRQOL and the changes of OHRQOL of oral cancer patients due to conventional RT is important in the countries like Sri Lanka where the majority of patients are treated with conventional RT. When addressing patient management decisions, health care professionals need to pay more attention regarding the perception and expectation of the patient. The knowledge of OHRQOL of these patients may provide the level and the most essential time period of support they need from the healthcare professionals. Further, it provides the success of the involvement of multidisciplinary healthcare team [19]. It will be important for the patients to know the changes take place in their OHRQOL while undergoing RT and after three months post RT. Up to now OHRQOL of the oral cancer patients undergoing RT has not been assessed in Sri Lanka. Considering all the facts mentioned, a prospective study was conducted to evaluate the OHRQOL and changes of OHRQOL among oral cancer patients who received RT alone or with chemotherapy.

## Methods

A prospective study was carried out at the National Cancer Institute (Apeksha Hospital) which is the main tertiary referral hospital for all cancers in Sri Lanka. The treatment plan for the patient was decided by the oncologists in consultation with the patient and the close family members regarding the best available treatment options, taking into account the patient’s age, stage and spread of cancer, co-morbidities, ability to withstand/tolerate the surgery, avoiding cosmetic imperfections and maintaining the functions. The study included only the oral cavity cancers as defined by the International Agency for Research on Cancer [20]. Therefore, lip, anterior two-thirds of the tongue, buccal mucosa, floor of mouth, hard palate, lower and upper alveolus and gingiva, and the retromolar trigone were taken as oral cancers in the study. From the medical records, oral cancer patients more than 18 years old whose initial treatment was decided as conventional RT alone or with chemotherapy were selected for this study as the aim was to assess the OHRQOL due to RT alone or with chemotherapy. Patients were excluded from the study if the initial treatment was surgery, RT as palliative treatment with small doses and Intensity Modulated RT using linear accelerator. Patients who were unable to participate in the interviews due to obvious cognitive and/or psychological impairment, who were followed up at private sector, those with evidence of brain metastases and participation in any other trials or studies interfering with the present study were also excluded. The sample size calculation suggested by Suresh and Chandrashekara was used in this study [21]. The final calculated sample was 90 after adding 20% to compensate for loss to follow up.

Oral cancer patients diagnosed within three months were recruited before the start of their radiation therapy. The waiting time for RT for the sample was less than three months after the pathological diagnosis. These patients were followed up for three months after completing the treatment. Informed written consents were obtained from the patients. Socio-demographic data were collected via face to face interview with the patients. The clinical data such as site of the cancer, stage of the cancer, treatment modality of the study participants was obtained from the medical records. Some of the patients had been referred to the dental clinic and had received dental treatments before the RT. Therefore, records were taken about the dental referrals and the treatments they had received. Patients completed the self-administered, modified EORTC QLQ-OH15 before RT (at baseline), during the last week of the RT and at three months post RT. Ethical clearance for the study was obtained from the Ethics Committee, Faculty of Medicine, University of Colombo, Sri Lanka (approval number - EC-15-200).

### Questionnaire

The European Organization for Research and Treatment of Cancer (EORTC) has developed the EORTC Quality of Life Questionnaire for Oral Health (EORTC QLQ-OH 15) to measure the OHRQOL in patients with any type of cancer [22]. The modified, translated and validated EORTC QLQ-OH15 questionnaire specifically to measure OHRQOL among oral cancer patients who receive RT alone or with chemotherapy in Sri Lanka, was used in this study. Thirteen variables were analyzed under three symptom domains namely *‘Eating problem’* (5 variables), *‘Gum and speech problem’* (4 variables) and *‘Soreness’* (3 variables) and a single symptom item *‘Teeth’.* In addition, there are two variables to assess the patients’ satisfaction on information they gained during treatment and two variables to assess OHRQOL among denture wearers. A high score represents a high level of symptoms and low level of OHRQOL [23].

### Statistical analysis

All three scales and the single-item in the modified EORTC QLQ-OH15 measures ranged in score from zero to 100. The distribution of the data was found to be non-parametric when assessed by normality tests. However, both the median and the mean values were presented to facilitate the comparison with the other studies. The significance of the changes of OHRQOL was tested using the Wilcoxon Signed Ranks Test and the p value < 0.05 was considered significant. Satisfaction of information received during treatment was presented separately as those results may be useful for the decision makers. Higher scores indicate higher satisfaction about the information they received. Dentures were not worn by any of the patients and therefore the two questions regarding the dentures were not analyzed. All the socio-demographic, clinical characteristics listed in Table 1 and baseline OHRQOL related to the domain were entered into the multiple linear regression model with the OHRQOL during last week of RT as the dependent variable.

## Results

Recruited study sample was 90 at baseline assessment and four patients (6.7%) and 15 patients (16.7%) were lost to follow up during the last week of RT and three months post RT respectively. The mean age of the sample was 59 (SD:+/-11) years. Of the sample, 88% were males and 91% were married. Almost 41% and 47% earned less than 15,000 and 15,000–30,000 Sri Lankan rupees respectively. When considering clinical characteristics, the anterior two-thirds of the tongue and buccal mucosa were the most common sites of oral cancer representing 40% and 22% of the sample respectively. 72% of patients were in stage III and stage IV and the majority (63%) was treated with RT with chemotherapy. Only 43% had received dental checkup or treatment before RT (Table 1).

The median of the ‘*Eating problem*’ domain was 20.0 (IQR: 66.7–33.3) at baseline. This domain increased to 100.0 (IQR: 86.9–100.0) during the last week of RT and decreased to 66.7 (IQR: 46.7–93.3) three months post RT. The medians for the *‘Soreness’* domain at the three time points were 11.1 (IQR: 0.00-33.3), 55.6 (IQR: 44.4–77.8) and zero respectively (Table 2). Of the sample, only seven patients had received the information regarding the possible dental and mouth problems before commencing RT. The patients’ satisfaction regarding the information they received about possible dental or mouth problems at three months post RT (mean: 41.8, SD: 19.5) was more than it was at the last week of RT (mean: 47.8, SD:17.7)(Table 3).

**Fig. 1:**
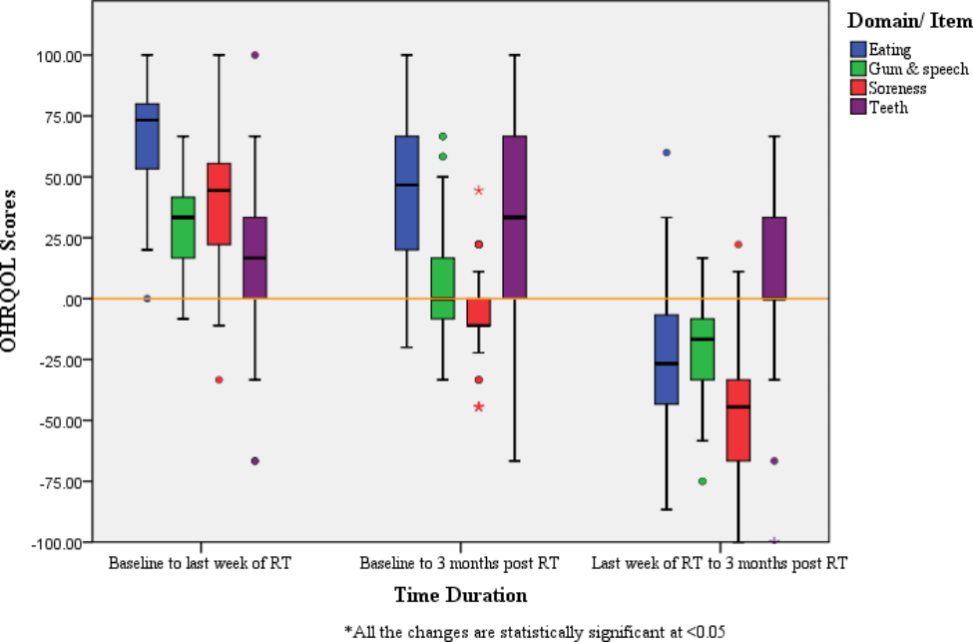
Comparison of Change of Modified EORTC QLQ-OH15 Scores (Change in OHRQOL) of the Sample

The median scores for change of symptoms from baseline to last week of the RT course for the ‘*Eating problem’, ‘Gum and speech problem’* and ‘*Soreness’* domains were 75.0, 33.3 and 44.4 respectively. It was 62.6 for the ‘*Teeth*’ item. The changes for all three domains and the item from baseline to last week of the RT, from baseline to three months post RT and from last week of RT to three months post RT were statistically significant (p < 0.05)(Fig. 1).

The OHRQOL related to *‘Eating problem’* domain during the last week of RT was influenced by marital status (p < 0.001) and the sites of metastasis (p < 0.05). The OHRQOL in relation to '*Gum & speech problem’* domain (p < 0.001) and *Teeth’* item (p < 0.01) at baseline were identified as influential factors for the OHRQOL during the last week of RT. OHRQOL related to '*Soreness*' domain was not influenced by any factor included in the regression model. Only the significant results were presented in the Table 4.

**Table 1:** Distribution of the Study Population by Socio Demographic and Clinical Characteristics (n = 90)

Characteristics	Frequency	Percentage (%)
**Age**		
35–49	14	15.5
50–69	61	67.8
>70	15	16.7
**Sex**		
Female	11	12.2
Male	79	87.8
**Marital status**		
Married	82	91.1
Unmarried	8	8.9
**Education level**		
Up to grade 5	28	31.1
Up to Ordinary Level	55	61.1
Up to Advanced Level	6	6.7
Diploma/ Degree	1	1.1
**Employment status**		
Unemployed	18	20.0
Self employed	55	61.1
Employed	9	10.0
Pensioner	8	8.9
Income*		
<15,000	37	41.1
15,000–30,000	42	46.7
> 30,000	11	12.2
**Site of the oral cancer**		
Lip	2	2.2
Anterior two-thirds of the tongue	36	40.0
Buccal mucosa	20	22.2
Floor of the mouth	12	13.3
Hard palate	5	5.6
Lower and upper alveolar ridge	1	1.1
Retromolar trigone	8	8.9
More than two sites**	6	6.7
**Stage**		
Early stage (stage I and II)	23	25.6
Late stage (stage III and IV)	65	72.2
Missing	2	2.2
**Metastasis**		
None	37	41.1
Lymph node	49	54.4
Systemic	2	2.2
Missing	2	2.2
**Treatment modality**		
Radiotherapy	33	36.7
Chemo-radiotherapy	57	63.3
**Other diseases**		
None	73	81.1
Any other disease ***	17	18.9
**Dental treatments received before RT**		
Yes****	39	43.3
No	49	54.4
Missing	2	2.3

*US$1 = LKR358.00

** Anterior two-thirds of the tongue and floor of mouth = 4, Hard palate, buccal mucosa and alveolus = 1, Buccal mucosa and floor of the mouth = 1

***Diabetes = 4, Hypertension = 4, Diabetes and hypertension = 2, Asthma = 2, Arthritis = 2, Gastritis = 2, Cerebrovascular diseases = 1

****Checkup only = 4, Scaling = 3, Restorations = 2, Extractions = 28, Scaling and extractions = 2

**Table 2:** Comparison of Modified EORTC QLQ-OH15 Scores of the Sample at Baseline, During Last Week of RT Course and Three Months After RT

Scores	Baseline (n = 90)		Last week of RT (n = 86)		Three months post RT (n = 75)	
	Mean (SD)	Median (IQR)	Mean (SD)	Median (IQR)	Mean (SD)	Median (IQR)
Eating problem	22.4 (19.1)	20.0 (6.7–33.3)	91.6 (13.3)	100.0 (86.9–100.0)	64.9 (28.2)	66.7 (46.7–93.3)
Gum and speech problem	13.5 (14.3)	8.3 (0.0-18.8)	44.2 (19.8)	50.0 (25.0- 58.3)	19.8 (22.8)	8.3 (0.0-33.3)
Soreness	14.6 (13.9)	11.1 (0.0-22.2)	56.9 (23.1)	55.6 (44.4–77.8)	9.8 (14.7)	0.0 (0.0-11.1)
Teeth	14.8 (20.1)	0.0 (0.0-33.3)	34.1 (29.8)	33.3 (0.0-66.7)	39.7 (33.2)	33.3 (0.0-66.7)

**Table 3:** Comparison of Scores of Satisfaction of the Information Received at Baseline, During Last Week of RT Course and Three Months After RT

Scores	Baseline (n = 7)		Last week of RT (n = 54)		Three months post RT (n = 61)	
	**Mean** **(SD)**	**Median** **(IQR)**	**Mean** **(SD)**	**Median (IQR)**	**Mean** **(SD)**	**Median (IQR)**
Satisfaction of information	66.7 (33.3)	66.7 (33.3–100.0)	41.8 (19.5)	33.3 (33.3–33.3)	47.8 (17.7)	33.3 (33.3–66.7)

**Table 4:** Multiple Linear Regression Analysis for the Predictors of OHRQOL During the Last Week of RT

Domain/Item		B	(β)	P Value	95% Confident Interval of B	
					Lower	Upper
Eating problem^a^	Constant	100.43		0.000	88.62	112.23
	Marital status	-17.00	− 0.374	0.001	-26.91	-7.1
	Sites of metastasis	6.00	0.241	0.031	0.57	11.43
Gum & speech^b^	Constant	34.02		0.000	28.91	39.13
	Baseline OHRQOL (Gum & Speech)	0.74	0.54	0.000	0.48	0.99
Teeth^c^	Constant	27.357		0.000	19.665	35.048
	Baseline OHRQOL (Teeth)	0.375	0.261	0.017	0.069	0.682

^a^R^2^=14%, Adjusted R^2^ = 12%

^b^R^2^=29%, Adjusted R^2^ = 28%

^c^R^2^=7%, Adjusted R^2^ = 6%

## Discussion

This was the first prospective study carried out among the oral cancer patients who receive RT alone or with chemotherapy to assess their OHRQOL and the changes of OHRQOL within three months post RT in Sri Lanka. The OHRQOL was assessed by using modified EORTC QLQ-OH15 questionnaire which was validated for the oral cancer patients who received RT alone or with chemotherapy in Sri Lanka [23]. The OHRQOL in oral cancer patients became extremely poor during the last week of RT compared to baseline due to RT alone or with chemotherapy. The OHRQOL had improved three months post RT from the last week of RT but had not returned to the baseline. OHRQOL during last week was influenced by baseline OHRQOL of the patient, marital status and the site of metastasis.

The majority of the study sample was consisted of males. This was anticipated as the most common cancer in males in Sri Lanka is oral cancer [3, 24]. Age of the most patients was between 50 and 69 years and a considerable percentage of the sample (16.7%) was consisted of those who were more than 70 years of age. This may be due to the fact that eligibility criteria of the present study when surgical management was not possible due to the old age, and RT or chemo-RT had been the treatment of choice. The commonest sites of the cancer were anterior 2/3rd of the tongue and buccal mucosa and at diagnosis most of the patients presented with late stage. Nevertheless, the commonest treatment modality was chemo-RT and a considerable amount of the sample was treated with RT only. These findings are connected to each other when the age of the patient and the late stage of the cancer were major factors for deciding the treatment modality [11]. In spite of knowing the fact that chemo-RT is better than RT alone for tumor control in advance stages, the physical fitness of the patient which could not be tolerated by the toxicity of systemic chemotherapy, may have influence the treatment decision taken by the Oncologist [8–10].

Most of the previous studies have assessed OHRQOL by using EORTC QLQ-H&N35 which was designed to measure health related quality of life among head and neck cancer patients. This may be due to the lack of a cancer specific tool to evaluate the OHRQOL in cancer patients until the recently developed EORTC QLQ-OH15 [22]. Even though EORTC QLQ-H&N35 was developed to assess health related quality of life in head and neck cancer patients, almost all the measurements were related to oral side effects except for a few scales namely ‘*Social contact’* and ‘*Less sexuality’*. Therefore, it is possible to interpret the results of EORTC QLQ-H&N35 scores as a measurement of OHRQOL. Braam et al. conducted a study among head and neck cancer patients and revealed significant changes in EORTC QLQ-H&N35 scores between baseline to six weeks after RT except for ‘*Social contact’ and ‘Less sexuality’* [25]. *‘Teeth’* and ‘*Open mouth’*. *‘Dry mouth’, ‘Sticky saliva’*, ‘*Problems of eating solid food*’ were hindered in the *‘Eating problem’* domain in the modified EORTC QLQ-OH15 which had shown significant changes from baseline to last week of RT. Similar results were shown in the most of the prospective studies done on OHRQOL [26, 27].

There is a tremendous amount of literature to support that oral symptoms become higher after RT even though the tools used were not specifically designed to measure OHRQOL among cancer patients [28–30]. Many studies have evaluated OHRQOL in patients with oral cancer using the Oral Health Impact Profile-14 and found a negative impact on OHRQOL after RT [31]. Santos et al. concluded that the oral health of head and neck cancer patients who had been treated with RT, deteriorated after RT with direct impact on their QOL [32]. In contrast to our study, OHRQOL at 3-3.5 months post RT, had recovered to the same level as baseline in a study conducted among Japanese head and neck cancer patients, but their results also showed rapid decrease during RT [33].

A prospective study conducted among head and neck cancer patients in Queensland, Australia to assess the changes of quality of life over time showed decreased scores in *‘Swallowing’, ‘Chewing’, ‘Speech’, ‘Taste’* and *‘Saliva’* domains after one month post treatment compared to baseline and the changes were statistically significant. The scores for the same domains had not returned to the baseline even six months post treatment in that study. Further, they observed statistically significant changes for the ‘*Chewing’ ‘Speech’* and *‘Taste’* domains from one month to six month post treatment [34]. The present study showed statistically significant results for the changes of all three domains namely ‘*Eating problem’*, ‘*Gum and speech problem’* and *‘Soreness’* and the item *‘Teeth’* from the last week of RT to baseline, three months post RT to baseline and three months post RT to the last week of RT. Another study conducted among oral cancer patients in Malaysia has shown the statistically significant changes in health related quality of life one and three months after treatment [35].

Shi et al. have shown that the married oral cancer patients are likely to have a better survival rate than unmarried patients [36] which was similar to our study. The support given by the spouse to early identification of the disease and to overcome the difficult situation during the course of treatment may have been the underline factors [37, 38]. In contrast, a study has revealed that the widowed head and neck cancer patients had a better OHRQOL in Brazil [39].

The level of OHRQOL before RT has significantly affected the OHRQOL during the last week of RT. Therefore, improving oral health before RT will enhance the quality of life of oral cancer patients after cancer treatments. Guidelines should be there to follow up by the clinicians to improve the OHRQOL of oral cancer patients who undergo RT, and timely referrals to the dentists, availability, collaboration, and coordination among interdisciplinary dental specialists found to be major factors when implementing the guidelines to provide oral care to the patients [40].

Educating the patients regarding the treatments and side effects has positive impact on reducing severity of side effects, health care outcomes, and improved quality of life [41, 42]. In present study information provided by the healthcare professionals on oral side effects before treatment was minimal. Only 2/3 of the sample had received such information after three months. However, the patients who had the knowledge about oral and dental side effects were not satisfied about the information they received. The health care professionals should consider this fact seriously as the patient’s positive attitudes help in managing the side effects effectively and improve their OHRQOL [42]. Our study revealed that the oral cancer patients treated with RT alone or with chemotherapy needed more support and care from the healthcare professionals before RT, throughout the RT course and until three months post RT and beyond to improve their ORQOL.

There were some limitations of this study. Some patients were recruited just before RT and others were recruited a few days/weeks before RT. Therefore, OHRQOL at baseline might be varied between patients. The study was confined only to three months after RT which allows short term evaluation of HRQOL affected by RT with or without chemotherapy. There might be selection bias as some of the oral cancer patients may seek treatments from private sector and other few government hospitals where patients are treated with RT. Details about medications and counselling were not recorded and the oral examination of the sample was also not performed during this study. However, high response rate, use of modified and validated questionnaires especially designed to capture OHRQOL of oral cancer patients were some strengths of this study. Furthermore, finding of this study was valid to reveal the OHRQOL of oral cancer patients who receive RT as the sample was a more homogenous group who had not undergone surgery and the treatment options were only confined to RT alone or with chemotherapy. The provision of oral health care for the patients undergoing RT is a mandatory requirement to improve the OHRQOL. The findings of this study may be useful for the health care providers and policy makers to develop a protocol to manage oral cancer patients who receive RT to improve their oral health prior to RT, during and post RT. Patients and their caregivers will be benefited by realizing the changes of OHRQOL that they have to face during and three months post RT and take prior precautions to minimize the effects.

## Conclusion

OHRQOL of oral cancer patients declined due to RT alone or with chemotherapy from baseline to last week of RT and improved three months after RT than the OHRQOL during last week of RT. Nevertheless, it had not come back to the baseline level, even after three months post RT. The changes in OHRQOL were statistically significant from baseline to last week of RT, from baseline and three months after RT, and from last week of RT to three months post RT. Baseline OHRQOL, marital status and sites of metastasis were the influential factors for the OHRQOL during the last week of RT.

## Data Availability

The datasets used and/or analysed during the current study are available from the corresponding author on reasonable request.

## List of Abbreviations

RT: Radiotherapy
OHRQOL: Oral health related quality of life
EORTC QLQ-OH15: European Organization for Research and Treatment of Cancer Quality of Life Questionnaire Oral Health Module
EORTC QLQ-H&N35: European Organization for Research and Treatment of Cancer quality of life head and neck questionnaire
SD: Standard deviation
IQR: Interquartile range
